# Seroprevalence of human T-lymphotropic virus HTLV and its associated factors in donors of a blood bank of Medellín-Colombia, 2014-2018

**DOI:** 10.1371/journal.pone.0221060

**Published:** 2019-08-12

**Authors:** Jaiberth Antonio Cardona-Arias, Carolina Vélez-Quintero, Olga Victoria Calle-González, Jennifer Florez-Duque, Juan Carlos Zapata

**Affiliations:** 1 School of Microbiology, University of Antioquia Faculty of Medicine, University Cooperative of Colombia, Medellín, Colombia; 2 School of Microbiology, University of Antioquia, Medellín, Colombia; 3 School of Microbiology, University of Antioquia, Medellín, Colombia; 4 Blood Bank, School of Microbiology, University of Antioquia, Medellín, Colombia; 5 Institute of Human Virology, University of Maryland School of Medicine, Baltimore, Maryland, United States of America; University of Cincinnati College of Medicine, UNITED STATES

## Abstract

**Background:**

Research on HTLV in Colombia is limited; despite being an endemic country there are few studies on the magnitude of this infection. The aim of this study was to determine the seroprevalence of HTLV I/II and its associated factors in donors to a blood bank of Medellín Colombia, 2014–2018.

**Methods:**

This is a cross-sectional study of 52,159 donors with a secondary information source. Seroprevalence of HTLV I/II was determined with its confidence interval and the population characteristics were described by frequency and summary measures. To explore the associated factors, Pearson’s Chi square test, Mann-Whitney U test, crude odds ratios were used and they were adjusted by logistic regression in SPSS 25.0.

**Results:**

88% of the population lived in the metropolitan area, 68.5% belonged to the University. 76.2% were altruistic donors (unpaid donors who did not donate to a specific patient). 24.5% were repetitive (paid) donors. 75% of the donors were under 41 years old. The seroprevalence of HTLV I/II was 0.176% (95% CI = 0.139% -0.213%), being statistically lower in repetitive donors and men.

**Conclusion:**

The seroprevalence of HTLV I/II infection in the studied blood bank is lower than that reported in other blood banks at the departmental and national levels. In Medellín, it was associated with the frequency of donation and gender, which is useful information for the hemovigilance programs of the city.

## Introduction

HTLV is the first human pathogenic retrovirus discovered in the laboratories of Dr. Gallo in 1979 [1]. With two strains of importance for human health, HTLV-I and HTLV-II, these retroviruses target T lymphocytic cells and are associated with multiple clinical manifestations [2]. The routes of transmission include mother-to-child, mainly through breastfeeding; sexual intercourse; and parenteral transmission through contaminated needles or transfusions from an infected donor [3].

After decades, HTLV-I chronic infection can trigger oncogenic diseases such as leukemia or T-cell lymphoma (ATL), characterized by its aggressiveness, resistance to treatment and high lethality. It can also generate neurological pathologies such as tropical spastic paraparesis (HAM/TSP); inflammatory diseases, for instance uveitis and osteoarthritis derived from the reactivity of monoclonal T lymphocytes; as well as the increase of opportunistic infections such as infective dermatitis and infection by *Strongyloides stercoralis* [4]. Those diseases related to HTLV-II include neurological conditions such as ataxia and chronic myelopathies [5].

All HTLV illnesses involve long and costly treatments that affect people’s quality of life. In the case of leukemias/lymphomas of T-lymphocytes, poor prognosis is usually caused by the disease per se and those disorders triggered by immunosuppression. In addition, HTLV infections also represent an important cost for health systems [6].

The number of infected people in the world ranges between 10 and 20 million [2]. The places with the highest prevalence of HTLV-I infection are southwestern Japan, sub-Saharan Africa, South America, the Caribbean area and some sub-regions of the Middle East and Australo-Melanesia [7]. Colombia is considered an endemic country for HTLV-I because the prevalence of the virus is estimated to be greater than 1% [8,9]. In the case of HTLV-II, its prevalence is higher among indigenous tribes in South America and in pygmies in Africa [10]. In the general population, the infection is usually detected in blood donors or by the presence of clinical symptoms; although more than 90% of infected patients are asymptomatic, with a long incubation time and a limited immunological window period during which HTLV sero-positivity could be detected [11,12].

Worldwide there are many studies on the sero-prevalence of HTLV in blood donors. In Japan from 2006 to 2007 a prevalence of 0.317% was reported in 1.2 million samples [13]. In Australia from 2000 to 2006 the prevalence was 0.3 per 100,000 samples [14], and in the United States in 2001 it was 9.6 for every 100,000 samples, or less than 0.01% [15].

In Costa Rica from 2002–2004, a study found, in 107 thousand samples, a reactivity of 0.54% by ELISA and a prevalence of 0.03% with the confirmatory test (WB) [16]. In Cuba from 1991 to 1996, sero-positivity evaluated by ELISA in 26,352 samples was 0.04%, with about 30% of infections acquired by blood transfusion [17]. In 9,425 samples collected in Santa Fe-Argentina from 1997 to 2002, the frequency HTLV-I/II infection was 0.11% [18]. These studies based their estimation on antibody measurement.

In Colombia there are several studies showing the exposure to the virus (reflected in the positivity of HTLV-I/II antibodies) in blood donors. In Antioquia (at the state level) HTLV-I/II antibodies were screened between 2001 and 2014 in 1.3 million donors showing a seroprevalence of 0.54% [19]. In Cali (at the city level) in 1997 the seroprevalence of HTLV was 0.69% and in the rest of the Cauca’s valley (at the state level) it was 0.75% [20]. In 2014, a prevalence of 0.26% was estimated for HTLV in 3,758 blood units in Bucaramanga (at the city level) [21]. In Medellín (at the city level), between 2014 and 2015 with 1,500 samples, a seroprevalence of 0.06% was registered [22]. Despite this background, the screening of HTLV in Colombian blood donors has become a routine or mandatory test only since 2014 [23], so recent studies are scarce and in many cases are not based on the totality of donors seen in blood banks.

In addition to the frequencies reported, other factors associated with HTLV infection should be considered in blood donors, for instance replacement donors, women, people with a high number of sexual partners, users of intravenous psychoactive substances, children of mothers with HTLV who were breastfed and young people between 18 and 33 years old [12,24,25]. However, the associated factors vary according to the study population, for instance, in other South American studies, it was reported that the prevalence is higher in men and in people between 34 and 64 years of age [25,26].

The information presented above shows the heterogeneity in the prevalence of HTLV in blood donors, as well as a low number of studies in Medellin (at the city level). Therefore, the objective of this study was to determine the seroprevalence of HTLV I/II and its associated factors in serum samples from donors to a blood bank in Medellín-Colombia, during the 2014–2018 period.

## Materials and methods

### Type and study subjects

We conducted a cross-sectional study of 52,159 donors to the blood bank of the School of Microbiology of the University of Antioquia in Medellín city, screened for HTLV I/II between the years 2014 and 2018.

The inclusion criteria were: age between 18 and 65 years, weight greater than 50 kg, hemoglobin greater than 12.5 g / dL in women and 13.5 g / dL in men, blood pressure between 100/60 mmHg and 180/90 mmHg.

The exclusion criteria were: having more than one sexual partner in the last 6 months, being or having been a sex worker or consumer of psychoactive substances intravenously, being pregnant or having given birth or suffered an abortion in the past months, having been ill in the last 15 days, having systemic autoimmune diseases such as lupus, previous infections transmissible by transfusion; having had piercings, tattoos, permanent makeup or acupuncture procedures in the last 12 months.

### Collection of information

We used a secondary source based on the records of the Hexabank software blood bank database license 1.28.30.50. From each donor, demographic information and the results of the HTLV I/II screening tests were collected, based on a chemiluminescent test that detects antibodies against HTLV-I /II with 99.5% specificity. All positive samples were confirmed by specific Western Blot for HTLV1 and HTLV 2 (band rgp 46-I, GD21, p19, p24, p26, p28, p32, p36, p53) with a sensitivity and specificity of 97%.

Validated laboratory methods were used to establish sero-positivity for HTLV I/II. Internal quality control of the Architect I200 immunoassay analyzer and external equipment of the Colombian National Institute of Health (INS) was carried out. The variables taken from the blood bank database were: age, gender, recruitment place (intramural means donations were from the blood bank of the hospital, and extramural means donations were from companies and universities), type of donation (altruistic donors were unpaid and donated to the blood bank voluntarily, and replenishment donors were directing their donation to replenish a particular patient), frequency of donation, type of extraction group, place of residence, and result of the screening and confirmatory test.

### Statistical analysis

The variables were described with summary measures and frequencies. Seroprevalence was determined as a proportion with a 95% confidence interval. To evaluate the assumption of normality in the quantitative variables, the Kolmogorov-Smirnov test was performed with Lilliefors correction. To explore the factors associated with the infection, Pearson Chi-square test was used for the nominal variables, Chi-square linear trend for the ordinal (age group), and Mann Whitney U for comparison with age. To adjust for the effects of confounding variables, odds ratios were adjusted using logistic regression. All analyses were performed in SPSS 25.0.

### Ethical aspects

The project complies with the guidelines of Helsinki, is a risk-free study according to Resolution 8430 of the Ministry of Health of Colombia 1993, and meets the provisions of Resolution No. 1995 of 1999 "By which standards are established for the Management of the Clinical History". In addition, the project has endorsement from the director of the Blood Bank, and all personally identifiable data were removed from the data set prior to access and analysis.

## Results

Of the study participants, 56% ranged between 22 and 41 years of age, 53.2% were women, 68.5% were extramural (from companies and universities), 58% lived in Medellín, 76.2% were altruistic donors, 45.6% were donors for the first time and 91.2% of the blood samples collected were whole blood (Table 1).

**Table 1:** Demographic description and some characteristics related to donation in the study.

Variables	Levels	n	%
**Age group**, years	18–20	8,870	17.0
	21–30	18,911	36.3
	31–40	10,683	20.5
	41–50	7,932	15.2
	51–65	5,763	11.0
**Gender**	Female	27,767	53.2
	Male	24,392	46.8
**Recruitment place**	Intramural ^a^	16,436	31.5
	Extramural ^b^	35,723	68.5
**Type of donation**	Altruistic ^c^	39,766	76.2
	Replenishment ^d^	12,393	23.8
**Frequency of donation**	First Time	23,763	45.6
	Not repetitive	15,617	29.9
	Repetitive	12,779	24.5
**Type of extraction grouped**	Total Blood	47,581	91.2
	Apheresis	4,578	8.8
**Place of residence**	Medellín	30,566	58.8
	Others of the Metropolitan Area	15,151	29.2
	Outside the Metropolitan Area	6,246	12.0

^a^ Intramural means donors were from the blood bank of the hospital

^b^ Extramural means donors were from companies and universities

^c^ Altruistic donors were unpaid and donated to the blood bank voluntarily

^d^ Replenishment donors were directing their donation to replenish a particular patient

The overall seroprevalence was 0.176% (95% CI = 0.139% -0.213%). Of the total of positive samples, the presence of HTLV 1 was confirmed in 12 donors, of HTLV 2 in four individuals, and in a woman of 21 years the presence of both was confirmed.

Age was not related to seropositivity (p> 0.05 U of Mann Whitney). Table 2 shows the specific seroprevalences, only statistical association was found with the frequency of donation (p = 0.02) and sex (p = 0.003).

**Table 2:** Comparison of the specific seroprevalence of HTLV according to the characteristics of the population.

Variable	Levels	n	Seroprevalence (%)
**Age group, years**	18–20	11	0.12
	21–30	38	0.20
	31–40	16	0.15
	41–50	14	0.18
	51–65	13	0.23
**Gender**	Female	63	0.23
	Male	29	0.12
**Recruitment place**	Intramural ^a^	31	0.19
	Extramural ^b^	61	0.17
**Type of donation**	Altruistic ^c^	68	0.17
	Replenishment ^d^	24	0.19
**Frequency of donation**	First Time	49	0.21
	Not repetitive	32	0.20
	Repetitive	11	0.09
**Type of extraction grouped**	Total Blood	88	0.18
	Apheresis	4	0.09
**Place of residence**	Medellín	52	0.17
	Others of the Metropolitan Area	27	0.18
	Outside the Metropolitan Area	13	0.21

^a^ Intramural means donors were from the blood bank of the hospital

^b^ Extramural means donors were from companies and universities

^c^ Altruistic donors were unpaid and donated to the blood bank voluntarily

^d^ Replenishment donors were directing their donation to replenish a particular patient

The seroprevalence of HTLV in women was 83% higher than in men, regardless of the frequency of donation. In first-time and non-repetitive donors, it was 2.25 and 2.31 times, respectively, that registered in repeat donors, independent of gender (Table 3).

**Table 3:** Association of the HTLV seroprevalence according to sex and frequency of donation.

Variables	OR Crude (IC 95%)	OR Adjusted (IC 95%)
**Gender** (Female / Male)	1.91 (1.23–2.97) **	1.83 (1.18–2.85) **
**Donation frequency**		
First time / Repetitive	2.40 (1.25–4.61) **	2.25 (1.17–4.33) *
Non-Repetitive / Repetitive	2.38 (1.20–4.73) **	2.31 (1.16–4.59) *

*p<0.05.

**p<0.01.

## Discussion

The groups with the highest frequency of donation were women, people under 41 and first-time donors and altruists; these results agree with reports from the National Institute of Health between 2014 and 2017. An exception was that the altruistic donation was lower than that reported in Antioquia (at the state level) with data of 82.3% in 2014 and 86.1% in 2017 [27,28]. This could be explained by the type of institution analyzed, because in donations from institutions not linked to clinics, the altruistic donations are more common, while in hospitals the replacement donors represent the vast majority [28].

Additionally, some demographic and cultural factors related to the type of donation predominant in each blood bank should be considered. A previous study showed a greater number of intramural donations from adults, while in the extramural donations the proportion of adolescent donors was higher (18–20 years). In addition, the donation by altruism or "social commitment" is more frequent from women, while in men the "own benefit” prevails [29,30].

The seroprevalence found in this investigation was lower than that reported from the departmental average of 0.30% and the national average of 0.54% [19]; although it approaches other studies with screening tests in high complexity blood banks in Medellín where they found a sero-positivity of 0.17% by ELISA [22]. In Cali, it was 0.24% by CMIA [31] and in Bogota 0.3% by ELISA [32]; while in Cartagena (at the city level) the highest seroprevalence was found with 3.1% by ELISA and chemiluminescence [33]. This heterogeneity accounts for the epidemiological particularities of exposure to the virus in the populations connected to the blood banks compared, as well as the need to conduct studies in each interest group given the high variability reported. Besides, the differences should not be attributed to the diagnostic tests because they are based on detection of antibodies with similar values of sensitivity and specificity, thus, reducing the probability of false negative and positive results.

In the study population, there was little association of HTLV infection with age, as in the blood bank of the Pablo Tobón Uribe Hospital located in the same city [22]. Most other studies of blood donors have shown differences in the proportion of infection according to age, with the largest group between 31 and 56 years of age in Bogotá, between 46 and 59 years of age in Cartagena and in people under 30 years of age in the Valle de Lili Foundation and in the national study with blood donors from endemic and non-endemic areas [31–34]. These discrepancies, in addition to accounting for the differential risk levels between age groups of several cities, could also be supported by the infection route, where high seroprevalence in younger population could be related to early acquisition of infection by vertical transmission and highest seroprevalences in adulthood through sexual contact [33].

In this study, the prevalence of HTLV-I/II was higher in women, which coincides with other studies in blood banks conducted in Medellín, Cali and Tumaco [22,31,35]. However, higher prevalences have been reported in men in Bogotá and Paraguay [32,36]. In the literature there are arguments regarding the greater or lesser transmission of men to women [37]. Possible explanations for the seroprevalence of one sex over the other could be linked to the behavior patterns of men and women in specific populations. Some authors have described that the higher risk in women can be attributed to a major specificity of the virus for this group, or the product of an increased risk of transmission from man to woman. For the related lentivirus, HIV-1, higher frequencies of sexual transmission to women have been attributed to higher female susceptibility [38]. At this point, the type of data collected in the blood banks of Medellín and the absence of analytical studies on HTLV in Colombia, prevent consolidating hypotheses and achieving profound explanations about the results found for the study population according to sex [39,40].

We also found higher seroprevalence in first-time donors and non-repetitive donors, which is consistent with reports in the literature that relate being a repetitive donor with lower HTLV-I /II seroprevalences in the United States and the United Kingdom. This has been explained by having better lifestyles and better quality of information from self-exclusion surveys in repetitive donors [41–43].

It must be remembered that donors must meet specific inclusion criteria and requirements that make them a low-risk group for infection, for this reason, the results shown are a good proxy for the level of exposure of the healthy general population in the area near the blood bank. However, it is important to highlight that HTLV infection in the general population may differ according to the behavior of each subgroup with different risk factors, such as the use of psychoactive substances or promiscuous behavior, from those without any risk factors. Additionally, according to guidelines of Colombian blood banks, viral RNA detection was not performed; in this sense, the study of the Demontis group concludes that "*HTLV-1 genomic RNA can be detected in the plasma of a minority of patients but not at a level or frequency to be useful clinically or diagnostically*. *Lack of transmission of HTLV-1 by plasma is due to the rare presence of HTLV-1 virions*, *regardless of any other factor*" [44].

Finally, the importance of this type of study should be highlighted, because it is aligned with the recommendations of experts from the Global Virus Network’s HTLV task force, HAM-net, and HTLV Aware suggesting as research priorities: epidemiological studies, mechanisms of HTLV-1 persistence, replication, pathogenesis, treatments, and prophylactic and therapeutic vaccines [45,46]. Particularly for blood banks, the results of this and other investigations demonstrate the need to increase the surveillance for HTLVI/II infections in all blood donors from endemic countries [47].

## Conclusion

In this study, the seroprevalence of HTLV I/II infection in the blood bank was lower than that reported in other banks at the departmental and national levels, and in the case of Medellín, it was only associated with the frequency of donation and gender, which is useful information for the hemovigilance programs of the city.

## Supporting information

S1 Database (SAV)

S2 Database (SAV)
